# Septic arthritis significantly increased the long-term mortality in geriatric patients

**DOI:** 10.1186/s12877-017-0561-x

**Published:** 2017-08-09

**Authors:** Chia-Jung Wu, Chien-Cheng Huang, Shih-Feng Weng, Ping-Jen Chen, Chien-Chin Hsu, Jhi-Joung Wang, How-Ran Guo, Hung-Jung Lin

**Affiliations:** 1 Department of Emergency Medicine, Chi-Mei Medical Center, 901 Zhonghua Road, Yongkang District, Tainan City, 710 Taiwan; 2 Department of Geriatrics and Gerontology, Chi-Mei Medical Center, Tainan, Taiwan; 3 Bachelor Program of Senior Service, Southern Taiwan University of Science and Technology, Tainan, Taiwan; 4 Department of Environmental and Occupational Health, College of Medicine, National Cheng Kung University, Tainan, Taiwan; 5 Department of Occupational Medicine, Chi-Mei Medical Center, Tainan, Taiwan; 6 Department of Healthcare Administration and Medical Informatics, Kaohsiung Medical University, Kaohsiung, Taiwan; 7 Palliative Care Center, Chi-Mei Medical Center, Tainan, Taiwan; 8 Department of Biotechnology, Southern Taiwan University of Science and Technology, Tainan, Taiwan; 9 Department of Medical Research, Chi-Mei Medical Center, Tainan, Taiwan; 10 Department of Occupational and Environmental Medicine, National Cheng Kung University Hospital, Tainan, Taiwan; 11 Department of Emergency Medicine, Taipei Medical University, Taipei, Taiwan

**Keywords:** Elderly, Geriatric, Mortality, Septic arthritis

## Abstract

**Background:**

The elderly are predisposed to septic arthritis (SA) because of the aging nature and increasing comorbidities. SA may in turn increase the long-term mortality in the geriatric patients; however, it remains unclear. We conducted this prospective nationwide population-based cohort study to clarify this issue.

**Methods:**

Using Taiwan National Health Insurance Research Database (NHIRD), we identified 1667 geriatric participants (≥ 65 years) with SA and 16,670 geriatric participants without SA matched at a ratio of 1:10 by age, sex, and index date between 1999 and 2010. A comparison of the long-term mortality between the two cohorts through follow-up until 2011 was performed.

**Results:**

Geriatric participants with SA had a significantly increased mortality than those without SA [Adjusted hazard ratio (AHR): 1.49, 95% confidence interval (CI): 1.34–1.66], particularly the old elderly (≥ 85 years, AHR: 2.12, 95% CI: 1.58–2.84) and males (AHR: 1.54, 95% CI: 1.33–1.79). These results were stated after adjustment for osteoarthritis, diabetes, gout, renal disease, liver disease, cancer, rheumatoid arthritis, systemic lupus erythematosus, alcoholism, and human immunodeficiency virus infection. The increased mortality risk was highest in the first month (AHR: 3.93, 95% CI: 2.94–5.25) and remained increased even after following up for 2–4 years (AHR: 1.30, 95% CI: 1.03–1.65). After Cox proportional hazard regression analysis, SA (AHR: 1.37, 95% CI: 1.20–1.56), older age (≥ 85 years, AHR: 1.79, 95% CI: 1.59–2.02, 75–84 years, AHR: 1.65, 95% CI: 1.53–1.78), male sex, diabetes, renal disease, liver disease, cancer, and gout were independent mortality predictors. There was no significant difference in the mortality for SA between upper limb affected and lower limb affected.

**Conclusions:**

This study delineated that SA significantly increased the long-term mortality in geriatric participants. For the increasing aging population worldwide, strategies for the prevention and treatment of SA and concomitant control of comorbidities are very important.

## Background

Aging issues are very important because the elderly (≥ 65 years old) are expected to increase rapidly from 6.2% of the world population in 1992 to 20% by 2050 [1]. In Taiwan, the geriatric population increased from 7% in 1993 to 12.5% in 2015 [2] and is expected to grow to 20% in 2025 [3]; Taiwan is one of the most rapidly aging countries in the world. The increasing geriatric population needs more medical healthcare resources, which reached 33.5% expenditure of Taiwan National Health Insurance in 2011; this percentage still rises [3].

Septic arthritis (SA) is a common joint infection [4] and is often caused by bacteria, viruses, or other less-common pathogens [4]. SA usually involves single and large joints, such as the knee joint, but many others may be involved [4]. In the United States, the incidence rate of SA is 0.01% in the general population annually; however, it increases to 0.07% in the high-risk groups such as those with rheumatoid arthritis or a prosthetic joint [5]. The predisposing factors for SA are as follows: (1) age > 80 years; (2) weak immune system due to diabetes, renal, and liver diseases, human immunodeficiency virus (HIV) infection, and use of immune-suppression drugs; (3) alcohol abuse; (4) cancer; (5) rheumatoid arthritis; (6) presence of prosthetic joint; (7) recent joint surgery; (8) skin infection; and (9) prior intra-articular corticosteroid injection [4–6].

Geriatric population is more vulnerable to SA because they have more risk factors than the younger population. The mortality for SA in geriatric patients is also higher than that in younger population due to delayed diagnosis and treatment, underlying comorbidities, and decreased physiological reserve [7]. Previous studies about geriatric SA are rare and focused on the identifications of the risk factors or treatments [8, 9]; however, the long-term mortality and mortality predictors after SA have never been clarified. Therefore, we conducted this nationwide population-based cohort based on Taiwan’s National Health Insurance Research Database (NHIRD) to determine the risk for long-term mortality in geriatric patients with SA as well as the mortality predictors.

## Methods

### Data sources

Taiwan launched a National Health Insurance program including almost all Taiwan’s citizens since March 1st, 1995 [10]. Large computerized databases containing the data of registration and claim diagnosis for reimbursement are derived from this system through the National Health Insurance Administration, Ministry of Health and Welfare, Taiwan and are maintained by the National Health Research Institutes, Taiwan; the databases are provided to scientists in Taiwan for research [10]. This study was based on the Longitudinal Health Insurance Database 2000 (LHID2000), which contains the entire original claim data of 1,000,000 beneficiaries enrolled in year 2000, randomly selected from the original NHIRD [10].

### Study design, participants, and definitions of the variables

**Fig. 1:**
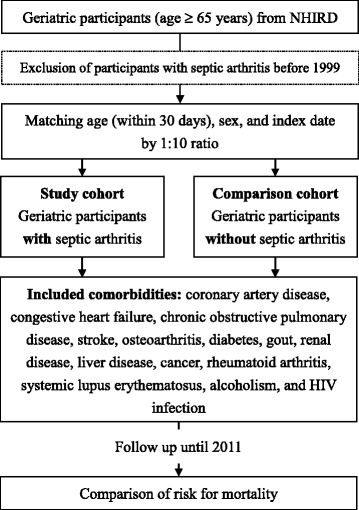
Flowchart of the study. NHIRD, National Health Insurance Research Database

Using LHID2000, we conducted a prospective nationwide population-based cohort study. We first identified all geriatric participants (age ≥ 65 years) and excluded those who had SA (ICD-9 code: 711.0) before 1999 (Fig. 1). We excluded participants with SA before 1999 to include new-onset SA only because this method could help us use the index date (i.e., the date of diagnosis for SA in the geriatric participants with SA) to match the comparison cohort and Cox proportional hazard regression to compare the risk for mortality. Next, we matched geriatric participants with and without SA between the period of January 1st, 1999 and December 31, 2010. These subjects were selected as the study cohort and the comparison cohort, respectively, matched at a ratio of 1:10 by age, sex, and index date. For stratified analysis, we categorized geriatric participants into three age subgroups: young elderly (65–74 years), moderately elderly (75–84 years), and old elderly (≥ 85 years), which is the most common criteria in the literatures [11, 12]. The underlying comorbidities that affect mortality included in this study were defined as follows: coronary artery disease (ICD-9 codes 410–414), congestive heart failure (ICD-9 code 428), chronic pulmonary obstructive disease (ICD-9 code 496), stroke (ICD-9 codes 436–438), osteoarthritis (ICD-9 code 715), diabetes (ICD-9 code 250), gout (ICD-9 code 274), renal disease (ICD-9 codes 582, 583, 585, 586, and 588), liver disease (ICD-9 codes 570–573), cancer (ICD-9 codes 140–208), rheumatoid arthritis (ICD-9 code 714), systemic lupus erythematosus (ICD-9 code 710.0), alcoholism (ICD-9 codes 291, 303), and HIV infection (ICD-9 codes 042–044). We also classified the affected areas as upper limbs (ICD-9 codes 711.01–711.04) and lower limbs (ICD-9 codes 711.05–711.07). Unspecified and multiple sites were excluded from the analysis concerning differences among affected sites due to the difficulty of classification. We followed up the participants until the end of 2011 to compare the all-cause mortality risk between both cohorts. According to the law in Taiwan, all citizens or people owning a residence permit are mandatory to participate in the National Health Insurance, and they must be dropped out of the National Health Insurance within 30 days after death. Therefore, we defined the death as the participant had the diagnosis of death or withdrew from the National Health Insurance. Stratified analyses by age subgroups and sex were performed to evaluate the effect modification of age and sex.

### Ethic statements

This study was approved by the Institutional Review Board at Chi-Mei Medical Center and conducted according to the Declaration of Helsinki. Because the LHID2000 used in this study consists of unidentifiable and secondary data released to the public for research, informed consent was waived. The waiver does not affect the rights and welfare of the participants.

### Statistical analysis

SAS 9.3.1 for Windows (SAS Institute, Cary, NC, USA) was used for all statistical analyses. In the comparison of age, sex, and underlying comorbidities between the two cohorts, we used Pearson chi-square tests for categorical variables and independent *t* test for continuous variables. Cox proportional hazard regression analysis with adjustment for coronary artery disease, congestive heart failure, chronic obstructive pulmonary disease, stroke, osteoarthritis, diabetes, gout, renal disease, liver disease, cancer, rheumatoid arthritis, systemic lupus erythematosus, and alcoholism was used to compare the mortality risk between the two cohorts. Kaplan-Meier curve and log-rank test for comparing mortality between participants with and without SA was also performed. Finally, we investigated the independent mortality predictor by Cox proportional hazard regression analysis. Significance level was set at 0.05 (two-tailed). Due to the simple covariate adjustment within Cox models may not fully adjust for such imbalance, and unmeasured confounder may exist [13]. Thus, we also conducted additional sensitivity analysis for the unmeasured confounder to strengthen the article base on the proposed method of Lin et al. (Appendix: Table 4).

## Results

The mean age in both cohorts was 74.6 years and the majority of geriatric participants were within the subgroup age 65–74 years (approximately 53%) (Table 1). The sex ratio was nearly equal in both cohorts. The common underlying comorbidities in the geriatric participants with SA were coronary artery disease (20.6%), congestive heart failure (8.4%), chronic pulmonary obstructive disease (17.3%), stroke (14.8%), osteoarthritis (32.3%), diabetes (28.6%), gout (17.4%), renal disease (9.9%), liver disease (9.5%), cancer (6.2%), rheumatoid arthritis (2.2%), and alcoholism (0.1%). There was no HIV infection in the participants. In the comparison of underlying comorbidities, geriatric participants with SA had significantly higher prevalence of coronary artery disease, congestive heart failure, chronic pulmonary obstructive disease, stroke, osteoarthritis, diabetes, gout, renal disease, liver disease, cancer, rheumatoid arthritis, and systemic lupus erythematosus than those without SA (all *p*-value <0.001).

**Table 1:** Demographic characteristics for geriatric participants with and without SA

Variable	With SA (*n* = 1667)	Without SA (*n* = 16,670)	*p*-value
Age (years, mean ± SD)	74.6 ± 6.7	74.6 ± 6.6	0.764
Age (years)			0.945
65–74	893 (53.6)	8964 (53.8)	
75–84	619 (37.1)	6197 (37.2)	
≥85	155 (9.3)	1509 (9.1)	
Sex			>0.999
Female	843 (50.6)	8430 (50.6)	
Male	824 (49.4)	8240 (49.4)	
Underlying comorbidity			
Coronary artery disease	343 (20.6)	2065 (12.4)	<0.001
Congestive heart failure	140 (8.4)	478 (2.9)	<0.001
Chronic pulmonary obstructive disease	288 (17.3)	1640 (9.8)	<0.001
Stroke	247 (14.8)	1484 (8.9)	<0.001
Osteoarthritis	539 (32.3)	1753 (10.5)	<0.001
Diabetes	477 (28.6)	2828 (17.0)	<0.001
Gout	290 (17.4)	752 (4.5)	<0.001
Renal disease	165 (9.9)	482 (2.9)	<0.001
Liver disease	158 (9.5)	717 (4.3)	<0.001
Cancer	103 (6.2)	680 (4.1)	<0.001
Rheumatoid arthritis	36 (2.2)	60 (0.4)	<0.001
Systemic lupus erythematosus	9 (0.5)	8 (0.1)	<0.001
Alcoholism	1 (0.1)	7 (0.04)	0.534
HIV infection	0	0	-

Data are expressed as mean ± SD or n (%)

*SA* septic arthritis, *HIV* human immunodeficiency virus

**Fig. 2:**
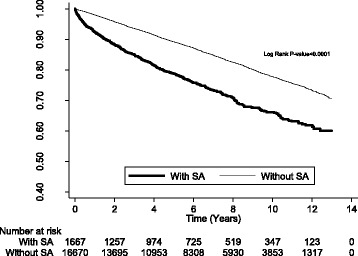
Kaplan-Meier curve and log-rank test between participants with SA and without SA (outcome = mortality)

Geriatric participants with SA had a significantly higher mortality risk than those without SA [adjusted hazard ratio (AHR): 1.39, 95% confidence interval (CI): 1.25–1.55] (Table 2). Older geriatric participants had higher mortality risk than the younger (AHRs in 65–74, 75–84, and ≥ 85 years old were 1.26, 1.44, and 1.86, respectively). Stratified analysis by sex showed there was a higher mortality risk in the male population than in female population. In the stratified analysis by the follow-up period, geriatric participants with SA had the highest mortality risk as compared with those without SA in the first 6 months after diagnosis (AHR: 3.38, 95% CI: 2.52–4.54). The impact of SA on mortality lasted until 1–2 years (AHR: 1.49, 95% CI: 1.11–1.98). Kaplan-Meier curve and log-rank test also showed that participants with SA had a lower survival than participants without SA during the follow-up (*p*-value < 0.001) (Fig. 2).

**Table 2:** Comparison of mortality risk between geriatric participants with and without SA using Cox proportional hazard regression analysis

Variable	With SA				Without SA				Crude HR (95% CI)	Adjusted HR^a^ (95% CI)
	*n*	Number of death	PY	Rate	*n*	Number of death	PY	Rate		
All	1667	430	9437.16	455.65	16,670	2545	104,188.3	244.27	1.87 (1.69–2.07)	1.39 (1.25–1.55)
Age (years)										
65–74	893	167	5612.95	297.53	8964	1075	60,096.6	178.88	1.67 (1.42–1.96)	1.26 (1.06–1.50)
75–84	619	196	3215.66	609.52	6197	1206	36,364.3	331.64	1.85 (1.59–2.15)	1.44 (1.23–1.68)
≥ 85	155	67	608.55	1100.98	1509	264	7727.47	341.64	3.15 (2.41–4.12)	1.86 (1.39–2.51)
Sex										
Female	843	191	5069.5	376.76	8430	1184	53,837.31	219.92	1.71 (1.47–2.00)	1.33 (1.13–1.56)
Male	824	239	4367.66	547.2	8240	1361	50,350.98	270.30	2.03 (1.77–2.32)	1.47 (1.27–1.70)
Follow-up period										
1–6 months	1667	86	795.7	1080.81	16,670	152	8166.58	186.12	5.73 (4.39–7.47)	3.38 (2.52–4.54)
6–12 months	1530	39	740.5	526.67	16,010	171	7807.51	219.02	2.40 (1.70–3.40)	1.61 (1.10–2.35)
1–2 years	1427	62	1344.36	461.19	15,185	332	14,472.93	229.39	2.01 (1.53–2.64)	1.49 (1.11–1.98)
2–4 years	1257	88	2225.73	395.38	13,695	571	24,604.46	232.07	1.70 (1.36–2.13)	1.20 (0.94–1.51)
4–6 years	974	61	1694.27	360.04	10,953	461	19,194.34	240.18	1.50 (1.15–1.96)	1.18 (0.90–1.56)
> 6 years	725	94	2636.6	356.52	8308	858	29,942.47	286.55	1.24 (1.01–1.54)	1.07 (0.86–1.34)

Data are expressed as mean ± SD or n (%)

*SA* septic arthritis, *AHR* adjusted hazard ratio, *CI* confidence interval, *PY* person-year

^a^Adjusted by coronary artery disease, congestive heart failure, chronic obstructive pulmonary disease, stroke, osteoarthritis, diabetes, gout, renal disease, liver disease, cancer, rheumatoid arthritis, systemic lupus erythematosus, and alcoholism

After Cox proportional hazard regression analysis, we found that SA (AHR: 1.23, 95% CI: 1.08–1.40), older age (≥ 85 and 75–84: AHR 2.18, 95% CI 1.93–2.47, and AHR 1.82, 95% CI 1.68–1.97, respectively), male sex (AHR: 1.23, 95% CI: 1.15–1.33), underlying comorbidities of congestive heart failure (AHR: 1.29, 95% CI: 1.10–1.51), chronic pulmonary obstructive disease (AHR: 1.49, 95% CI: 1.35–1.65), stroke (AHR: 1.66, 95% CI: 1.50–1.84), diabetes (AHR: 1.69, 95% CI: 1.55–1.83), gout (AHR: 1.25, 95% CI: 1.10–1.43), renal disease (AHR: 2.29, 95% CI: 2.00–2.62), liver disease (AHR: 1.51, 95% CI: 1.32–1.73), cancer (AHR: 1.74, 95% CI: 1.51–2.01), and rheumatoid arthritis (AHR: 1.55, 95% CI: 1.02–2.34) were independent mortality predictors (Table 3). Participants with SA affected in lower limbs had a lower mortality than in upper limbs.

**Table 3:** Independent predictors for mortality in the total participants using Cox proportional hazard regression analysis

Variable	Crude HR (95% CI)	AHR (95% CI)*	*p*-value†
Cohort			
With SA	1.87 (1.69–2.07)	1.23 (1.08–1.40)	0.002
Without SA	1	1	
Age (years)			
65–74	1	1	
75–84	1.90 (1.76–2.05)	1.82 (1.68–1.97)	<0.001
≥ 85	2.14 (1.89–2.41)	2.18 (1.93–2.47)	<0.001
Sex			
Female	1	1	
Male	1.26 (1.17–1.35)	1.23 (1.15–1.33)	<0.001
Underlying comorbidity			
Coronary artery disease	1.48 (1.35–1.63)	1.10 (0.99–1.21)	0.069
Congestive heart failure	2.54 (2.20–2.94)	1.29 (1.10–1.51)	0.001
Chronic pulmonary obstructive disease	2.09 (1.90–2.29)	1.49 (1.35–1.65)	<0.001
Stroke	2.21 (2.01–2.44)	1.66 (1.50–1.84)	<0.001
Osteoarthritis	1.07 (0.97–1.19)	0.99 (0.89–1.11)	0.929
Diabetes	1.94 (1.79–2.10)	1.69 (1.55–1.83)	<0.001
Gout	1.62 (1.42–1.84)	1.25 (1.10–1.43)	0.001
Renal disease	3.76 (3.31–4.27)	2.29 (2.00–2.62)	<0.001
Liver disease	1.85 (1.62–2.11)	1.51 (1.32–1.73)	<0.001
Cancer	1.90 (1.65–2.18)	1.74 (1.51–2.01)	<0.001
Rheumatoid arthritis	1.86 (1.23–2.80)	1.55 (1.02–2.34)	0.039
Systemic lupus erythematosus	0.96 (0.31–2.99)	0.62 (0.20–1.93)	0.411
Alcoholism	0.74 (0.10–5.24)	0.81 (0.11–5.73)	0.829
Affected area			
Upper limbs (*n* = 109)	1.00	1.00	
Lower limbs (*n* = 585)	0.88 (0.62–1.25)	0.69 (0.57–0.84)	<0.001

*SA* septic arthritis, *HR* hazard ratio, *CI* confidence interval, *PY* person-year

*Adjusted by coronary artery disease, congestive heart failure, chronic obstructive pulmonary disease, stroke, osteoarthritis, diabetes, gout, renal disease, liver disease, cancer, rheumatoid arthritis, systemic lupus erythematosus, and alcoholism

†For AHR

## Discussion

This prospective nationwide population-based cohort study revealed that geriatric participants with SA had a significantly higher mortality risk than those without SA. The common underlying comorbidities in geriatric participants with SA were osteoarthritis, diabetes, coronary artery disease, chronic pulmonary obstructive disease, gout, stroke, renal disease, liver disease, congestive heart failure, cancer, and rheumatoid arthritis. SA impacted the mortality risk more markedly in the first 6 months after the diagnosis and its effect lasted until 1–2 years. The mortality in older geriatric participants was affected by SA more frequently than that in younger geriatric participants. Male sex, older age, congestive heart failure, chronic pulmonary obstructive disease, stroke, diabetes, gout, renal disease, liver disease, cancer, and rheumatoid arthritis were also independent mortality predictors.

Advancing age is itself a risk factor and a mortality predictor for SA [14]. A single hospital-based study for SA reported that 61.4% of patients were >60 years and 12.5% of patients were ≥80 years [14]. The hospital mortality rate increased with age: 0.7% of patients <60 years, 4.8% of those 60–79 years, and 9.5% of those ≥80 years [14]. Another study reported that despite surgical treatment in the geriatric patients with SA, the complications were still high: 38% had osteomyelitis, 14% had secondary osteoarthritis, and 19% showed mortality due to sepsis [15]. In addition to the decreased immunity and increased comorbidities in the geriatric patients, the other explanation was that the diagnosis of infection in the elderly is more difficult due to the atypical manifestations [7]. Positive outcome needs an early diagnosis and treatment [7, 15]. The explanation for increased long-term mortality in geriatric participants with SA is multi-factorial. One of them is the dysfunction of the joint despite proper treatment, which results in subsequent disability of daily activity [16]. A study reported that severe activity of daily life (ADL) limitations and receiving ADL assistance significantly increased the subsequent mortality [odds ratio (OR): 19.75, 95% CI 9.81–39.76 and OR: 16.57, 95% CI 8.39–32.73, respectively] [17]. Another study reported that the disability for at least one ADL item predicted in-hospital death in the admitted geriatric patients (OR: 2.16, 95% CI: 1.55–2.99) [18]. A population-based study in the Netherlands reported that there was an increased mortality risk in the disabled population than in the non-disabled population [19]. Severe disability may has an independent effect for mortality despite that the risk factors preceding the disability could explain the difference in mild disability [19].

Inflammation after SA or its complications is also a possible mechanism for increasing the mortality [20]. A study about acute-care hospitalized elderly patients reported that C-reactive protein (CRP) ≥ 30 mg/l predicted mortality (OR: 3.72, 95% CI: 1.34–10.31) [18]. SA may result in subsequent osteomyelitis [15] or chronic SA, which may cause a chronic inflammation and increase long-term mortality [21]. A nationwide population-based cohort study reported that geriatric participants with chronic osteomyelitis had a significantly higher mortality risk than those without chronic osteomyelitis [incidence rate ratio (IRR): 2.29, 95% CI: 2.01–2.59] [21]. The mortality risk was highest in the first month (IRR: 5.01, 95% CI: 2.02–12.42) and still higher even after 6 years (IRR: 1.53, 95% CI: 1.13–2.06) of follow-up [21]. This study did not analyze the cause of chronic osteomyelitis; however, it did provide us an insight into the effect of chronic osteomyelitis, a common complication after SA. In addition to ADL limitations and inflammation, the possible explanations for highest mortality risk in the first 6 months after the diagnosis are disseminated infection, acute renal failure, and cardiopulmonary failure [22].

We showed that joints of the lower limbs were more affected than those of the upper limbs (*n* = 585 vs. *n* = 109), which was consistent with previous studies that showed that large joints in the lower limbs, such as knee and hip, were more commonly involved than small joints [14–16, 22]. The present study showed that SA in the lower limbs had a lower mortality than in upper limbs. However, a previous study reported that SA involving the hip or shoulder predicted poor outcome [23], which suggests that the comparison of outcome between the upper or lower limbs warrants more study in the future.

We showed that male sex, older age, congestive heart failure, chronic pulmonary obstructive disease, stroke, diabetes, gout, renal disease, liver disease, cancer, and rheumatoid arthritis also predicted mortality; however, systemic lupus erythematosus, a risk factor for SA [24], did not predict mortality. It suggests that we should treat SA as well as control other comorbidities simultaneously to decrease the subsequent mortality.

The strength of this study was that it clarified the long-term mortality and independent mortality predictors in geriatric participants with SA, which remained unclear. Despite its strength, this study had some limitations. First, the NHIRD contains no information on the severity of SA, some important laboratory data such as pathogens of blood culture or synovial fluid, and participants’ social economic status and post-diagnosis health care conditions (e.g., received good or bad quality health care? stay at home or in hospital or in nursing home?); therefore, we were unable to evaluate the severity association between them. Studies with more detail information are needed for the causal relationship between mortality and SA. Second, we did not identify the drug use, definite infected joints, and the relationship with previous surgery or prosthesis. Third, categorization of age is more convenient for clinical use; however, it may loss some information. Fourth, although our study was a nationwide population-based study, the result may not be applied to other nations due to the differences in race, culture, and environment.

## Conclusions

This prospective nationwide population-based cohort study showed that long-term mortality was significantly higher in geriatric participants with SA than in those without SA. The influence was highest in the first 6 months after diagnosis and lasted until 1–2 years. The impact of SA on mortality was more pronounced in the older geriatric participants than in the younger geriatric participants. In addition to SA, male sex, older age, congestive heart failure, chronic pulmonary obstructive disease, stroke, diabetes, gout, renal disease, liver disease, cancer, and rheumatoid arthritis were independent mortality predictors. For the increasing aging population worldwide, strategies for the prevention and treatment of SA and concomitant control of comorbidities are very important.

## Appendix

**Table 4:** Sensitivity analysis for the residual confounding effects of unmeasured variable after adjusting for measured covariates

P_0_							
r	P_1_	0	0.1	0.2	0.3	0.4	0.5
2	0	1.23	1.353	1.476	1.599	1.722	1.845
		(1.080,1.400)	(1.188,1.540)	(1.296,1.680)	(1.404,1.820)	(1.512,1.960)	(1.620,2.100)
	0.2	1.025	1.127	1.23	1.333	1.435	1.537
		(0.900,1.167)	(0.990,1.283)	(1.080,1.400)	(1.170,1.517)	(1.260,1.633)	(1.350,1.750)
	0.4	0.879	0.966	1.054	1.142	1.23	1.318
		(0.771,1.000)	(0.849,1.100)	(0.926,1.200)	(1.003,1.300)	(1.080,1.400)	(1.157,1.500)
	0.6	0.769	0.846	0.922	0.999	1.076	1.153
		(0.675,0.875)	(0.743,0.962)	(0.810,1.050)	(0.878,1.137)	(0.945,1.225)	(1.013,1.312)
	0.8	0.683	0.752	0.82	0.888	0.957	1.025
		(0.600,0.778)	(0.660,0.856)	(0.720,0.933)	(0.780,1.011)	(0.840,1.089)	(0.900,1.167)
	1	0.615	0.676	0.738	0.8	0.861	0.922
		(0.540,0.700)	(0.594,0.770)	(0.648,0.840)	(0.702,0.910)	(0.756,0.980)	(0.810,1.050)
6	0	1.23	1.845	2.46	3.075	3.69	4.305
		(1.080,1.400)	(1.620,2.100)	(2.160,2.800)	(2.700,3.500)	(3.240,4.200)	(3.780,4.900)
	0.2	0.615	0.922	1.23	1.537	1.845	2.153
		(0.540,0.700)	(0.810,1.050)	(1.080,1.400)	(1.350,1.750)	(1.620,2.100)	(1.890,2.450)
	0.4	0.41	0.615	0.82	1.025	1.23	1.435
		(0.360,0.467)	(0.540,0.700)	(0.720,0.933)	(0.900,1.167)	(1.080,1.400)	(1.260,1.633)
	0.6	0.307	0.461	0.615	0.769	0.923	1.076
		(0.270,0.350)	(0.405,0.525)	(0.540,0.700)	(0.675,0.875)	(0.810,1.050)	(0.945,1.225)
	0.8	0.246	0.369	0.492	0.615	0.738	0.861
		(0.216,0.280)	(0.324,0.420)	(0.432,0.560)	(0.540,0.700)	(0.648,0.840)	(0.756,0.980)
	1	0.205	0.307	0.41	0.513	0.615	0.718
		(0.180,0.233)	(0.270,0.350)	(0.360,0.467)	(0.450,0.583)	(0.540,0.700)	(0.630,0.817)
10	0	1.23	2.337	3.444	4.551	5.658	6.765
		(1.080,1.400)	(2.052,2.660)	(3.024,3.920)	(3.996,5.180)	(4.968,6.440)	(5.940,7.700)
	0.2	0.439	0.835	1.23	1.625	2.021	2.416
		(0.386,0.500)	(0.733,0.950)	(1.080,1.400)	(1.427,1.850)	(1.774,2.300)	(2.121,2.750)
	0.4	0.267	0.508	0.749	0.989	1.23	1.471
		(0.235,0.304)	(0.446,0.578)	(0.657,0.852)	(0.869,1.126)	(1.080,1.400)	(1.291,1.674)
	0.6	0.192	0.365	0.538	0.711	0.884	1.057
		(0.169,0.219)	(0.321,0.416)	(0.472,0.612)	(0.624,0.809)	(0.776,1.006)	(0.928,1.203)
	0.8	0.15	0.285	0.42	0.555	0.69	0.825
		(0.132,0.171)	(0.250,0.324)	(0.369,0.478)	(0.487,0.632)	(0.606,0.785)	(0.724,0.939)
	1	0.123	0.234	0.344	0.455	0.566	0.676
		(0.108,0.140)	(0.205,0.266)	(0.302,0.392)	(0.400,0.518)	(0.497,0.644)	(0.594,0.770)

## Abbreviations

ADL: activity of daily life
AHR: adjusted hazard ratio
CI: confidence interval
CRP: C-reactive protein
HIV: human immunodeficiency virus
ICD: International Classification of Diseases
IRR: incidence rate ratio
LHID: Longitudinal Health Insurance Database
NHIRD: National Health Insurance Research Database
OR: odds ratio
SA: septic arthritis
